# Variations in Screening Quality in a Federal Colorectal Cancer Screening Program for the Uninsured

**DOI:** 10.5888/pcd16.180452

**Published:** 2019-05-30

**Authors:** Marion R. Nadel, Janet Royalty, Djenaba Joseph, Tanner Rockwell, William Helsel, William Kammerer, Simone C. Gray, Jean A. Shapiro

**Affiliations:** 1 Division of Cancer Prevention and Control, Centers for Disease Control and Prevention, Atlanta, Georgia; 2 Information Management Services Inc, Calverton, Maryland

## Abstract

**Introduction:**

Screening can decrease colorectal cancer incidence and mortality and is recommended in clinical practice guidelines. Poor quality of colorectal cancer screening can negate the benefit of screening. The objective of this study was to assess the quality of screening services provided by the Centers for Disease Control and Prevention’s Colorectal Cancer Control Program from July 2009 through June 2015.

**Methods:**

We collected data from the program’s 29 grantees, funded to provide colorectal cancer screening and diagnostic services to asymptomatic, low-income, and underinsured or uninsured adults aged 50 to 64. We collected data on the dates and results of all screening and diagnostic tests and, for colonoscopies, on whether the cecum was reached, whether bowel preparation was adequate, and endoscopists’ recommendations for the next test.

**Results:**

Overall, 82.9% (range among grantees, 50.0%–97.2%) of positive FOBTs/FITs were followed up by colonoscopy; 95.2% of colonoscopies occurred within 180 days of the positive stool test. Cecal intubation rates ranged among grantees from 94.2% to 100%. Adenoma detection rates met recommended threshold levels for almost all grantees. Recommendations for rescreening and surveillance intervals deviated from guidelines in both directions. Of clients with normal colonoscopies, 85.3% (range, 37.7%–99.7%) were told to return in 10 years, as recommended in national guidelines. Of clients with advanced adenomas, 55.2% (range, 20.0%–84.6%) were told to return in 3 years as recommended, 25.4% (range, 3.8%–56.6%) in 5 or more years, and 18.6% (range, 0%–47.2%) in less than 3 years.

**Conclusion:**

Although overall screening quality was good, it varied considerably. Ongoing monitoring to identify performance problems is essential for all colorectal cancer screening activities, so that efforts designed to improve performance can be targeted to individual clinicians.

**Summary**

**What is already known on this topic?**

Colorectal cancer screening can be of little value if performed poorly. Problems with screening implementation are well documented, and quality indicators have been defined for routine monitoring in clinical practice.

**What is added by this report?**

In the Center for Disease Control and Prevention’s Colorectal Cancer Control Program, overall screening quality was good. However, even with the funding and oversight provided by this federal program, we found that quality indicators varied and some grantees fell short of desired levels.

**What are the implications for public health practice?**

Ongoing quality monitoring to identify performance problems is essential. Efforts to increase screening uptake need to be accompanied by efforts to assess and improve quality.

## Introduction

Screening can decrease colorectal cancer (CRC) incidence and mortality and is recommended in clinical practice guidelines (1). However, only two-thirds of adults aged 50 to 75 were up-to-date with CRC screening in 2016, well below the target set by the National Colorectal Cancer Roundtable’s initiative,“80% by 2018” (2,3). Screening can be of little value, however, if performed poorly. Because of well-documented variability in the quality of screening implementation (4–7), efforts to assess and improve screening quality need to accompany efforts to increase screening uptake. Common implementation problems include failure to follow up positive stool tests with colonoscopy, wide variation in the ability of endoscopists to detect adenomas, and recommended rescreening or surveillance intervals that do not comply with national guidelines. Improved colonoscopy quality has become a priority of professional societies. Quality indicators were defined for routine monitoring in clinical practice, and colonoscopy registries were developed to facilitate the process (5,8). Payment to providers increasingly incorporates quality assessment (9). Monitoring quality can lead to targeted improvement activities.

In 2005, the Centers for Disease Control and Prevention (CDC) launched the Colorectal Cancer Screening Demonstration Program (CRCSDP) at 5 sites to assess the feasibility of providing CRC screening, diagnostic, and surveillance services to low-income persons (10). An assessment of screening quality in the CRCSDP showed the need for improvement in several areas, such as the follow-up of positive stool tests and recommendations for rescreening and surveillance intervals (11).

After the CRCSDP, CDC established the Colorectal Cancer Control Program (CRCCP) at 29 sites in the United States (12). The objective of this study was to assess the quality of services provided in this expanded program from July 2009 through June 2015.

## Methods

**Figure 1:**
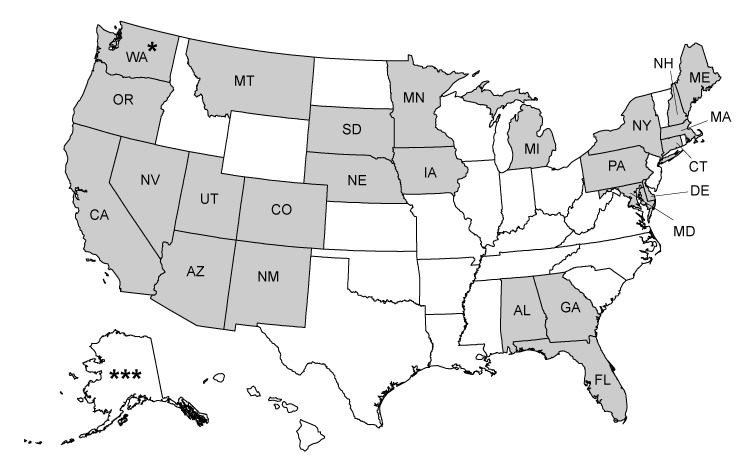
Twenty-nine grantees in the Centers for Disease Control and Prevention’s Colorectal Cancer Control Program, 2009–2015. Shading indicates a grantee state. An asterisk indicates a tribal grantee.

From 2009 to 2015, CDC provided CRCCP funding to grantees in 25 states and 4 tribal organizations for CRC screening, surveillance, and diagnostic services to asymptomatic, low-income, and underinsured or uninsured adults aged 50 to 64 (Figure 1). Details on the CRCCP are provided elsewhere (12). Our analyses consisted of data collected from 28 grantees, identified herein by randomly assigned numbers; we excluded 1 grantee from our analyses because a high percentage of its client records had missing information. 

As part of the program, grantees were permitted to use any screening tests recommended by the US Preventive Services Task Force in 2008: colonoscopy, guaiac fecal occult blood tests (FOBT), fecal immunochemical tests (FIT), or flexible sigmoidoscopy (13). For each CRCCP client, grantees collected a standardized set of CRC clinical data elements (CCDEs): age, sex, personal history of colorectal polyps or cancer, self-report of any prior CRC screening before CRCCP enrollment (but not information on which tests they had), and family history of CRC. Each grantee defined its own criteria for increased risk based on available guidelines (14).

For each test, grantees recorded the date of the test, the reason for test (screening, surveillance, or diagnostic), and the results of the test. For each colonoscopy, the CCDEs specified whether the cecum was reached (the **cecum** marks the beginning of the large intestine and a complete examination is one in which the scope progresses all the way to the **cecum**), whether the endoscopist considered the bowel preparation adequate, whether a polypectomy was performed, the number of polyps found, the worst histology among all polyps removed, and the clinician’s recommendation for which test the client should have next and when. Because endoscopy reporting was not standardized, grantee staff members occasionally converted the terms found in reports to fit the categories specified in the CCDEs.

Data quality was monitored at multiple steps. Before biannual submission of data to CDC, grantees checked their data with editing software provided by CDC to identify invalid values, missing fields, and cross-field inconsistencies. The data were then checked by CDC, and standard quality reports were produced. Calls were held with grantees to resolve identified discrepancies and discuss problem areas.

We tabulated data from the CCDEs for the period July 2009 through June 2015 on tests received by clients who did not report having CRC symptoms. We considered clients to be at average risk of CRC if they did not report any personal history of CRC or adenomas and were not at increased risk because of reported family history. We classified a colonoscopy as complete if the cecum was reached, bowel preparation was adequate, polyps were completely removed, and the procedure was not terminated early. All other colonoscopies were classified as incomplete. Only complete colonoscopies were included in our analyses of rescreening and surveillance recommendations and of adenoma detection rates (ADRs).

### Statistical analysis

We computed several quality indicators related to stool testing (completeness and timeliness of follow-up of positive tests) and to colonoscopy (cecal intubation rate, adequacy of bowel preparation quality, appropriateness of recommendations for rescreening and surveillance intervals after colonoscopy, and ADR). We compared our findings to targets established by CDC for the CRCCP and to targets established by various professional organizations (4,5,14–19). 

We computed the ADR as the percentage of colonoscopies in which at least 1 adenoma was reported. An adenoma is a type of polyp that may be a precursor lesion to colorectal cancer. Because adenoma prevalence varies by age and sex, we computed sex-specific ADRs for clients aged 50 years or older to allow comparison with published rates. For ADRs and the clinician’s recommendation after colonoscopy, we limited our analysis to data on the first screening colonoscopy received by each client in the CRCCP. We limited our analysis of clinicians’ follow-up recommendations to average-risk clients. We computed the cecal intubation rate as the percentage of colonoscopies in which the cecum was reached. 

We tabulated combined data on all 28 grantees. In addition, we tabulated data for each grantee separately; for these data, we tabulated data only for grantees with at least 30 data points. We computed ADRs only for grantees that had at least 30 clients in the categories *sex* and *reason for test*. Although the reliability of rates based on small numbers may be low and may not accurately measure performance, we chose to calculate grantee-specific data based on a low cutoff so that we could present data from as many grantees as possible. Rates based on small numbers should be interpreted cautiously.

For all analyses, we used SAS version 9.4 (TS1M5) (SAS Institute Inc).

## Results

Some grantees provided colonoscopy as the primary screening test, some provided stool tests (FOBT or FIT), and others used both types of test (Table 1). For tests used for screening, the ratio of stool tests to endoscopy was approximately 3:2.

**Table 1:** Number of Tests Provided in the Colorectal Cancer Control Program, by Grantee, 2009–2015^a^

Grantee Identifier^b^	Screening				Diagnostic Colonoscopy
	FOBT	FIT	Flexible Sigmoidoscopy	Colonoscopy	
1	0	0	0	1,700	0
2	0	2,478	2	571	116
3	11	188	1	2,206	1
4	6	5,350	0	285	290
5	0	0	4	1,800	0
6	0	0	0	857	0
7	0	74	492	0	5
8	0	148	8	2,311	6
9	4	7	2	1,715	10
10	0	3,032	0	4	265
11	3,980	0	2	593	188
12	0	1,166	0	217	50
13	4,360	2,657	3	915	604
14	34	400	2	1,928	37
15	3,098	3	0	967	103
16	840	415	1	309	35
17	0	1,011	0	26	36
18	0	468	0	721	19
19	0	0	0	1,754	0
20	12	0	0	978	0
21	0	2,003	1	228	139
22	0	0	0	275	0
23	0	12	2	1,352	0
24	0	116	0	0	6
25	12	318	1	538	31
26	779	504	10	2,199	138
27	0	1,666	3	242	368
28	137	3,241	0	167	202
All 28 grantees	13,273	25,257	534	24,858	2,649

Abbreviations: FIT, fecal immunochemical test; FOBT, guaiac fecal occult blood test.

a The Centers for Disease Control and Prevention provided Colorectal Cancer Control Program funding to grantees in 25 states and 4 tribal organizations for colorectal cancer screening, surveillance, and diagnostic services for underinsured or uninsured asymptomatic, low-income adults aged 50–64. One grantee was excluded from analysis because of missing data.

b Grantees identified by randomly assigned numbers.

### Stool tests

#### Positivity rate

**Figure 2:**
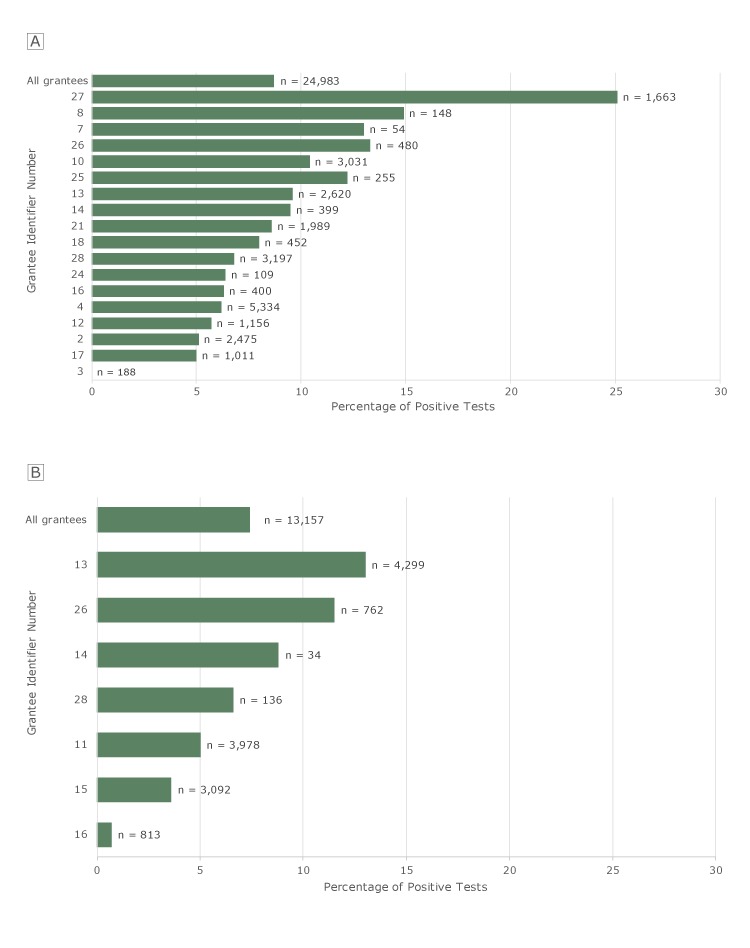
Positivity rates for FITs and FOBTs among clients aged ≥50, by grantee, Colorectal Cancer Control Program, 2009–2015. N’s indicate number of tests. A, FIT positivity rates. Only the 18 grantees that recorded ≥30 FITs are shown individually. “All grantees” refers to all grantees, including grantees that had <30 tests. B, FOBT positivity rates. Only the 7 grantees that recorded ≥30 FOBTs are shown individually. “All grantees” refers to all grantees, including the grantees that had <30 tests. Tests for which results were not known were excluded from these analyses. Abbreviations: FIT, fecal immunochemical test; FOBT, fecal occult blood test.

Of the 24,983 FITs completed by clients at 21 grantees, 8.7% were positive. Among the 18 grantees with at least 30 tests, positivity rates ranged from 0% to 25.1% (Figure 2A). Of the 13,157 FOBTs completed by clients at 12 grantees, 7.4% were positive. Among the 7 grantees with at least 30 tests, positivity rates ranged from 0.7% to 13.0% (Figure 2B). 

**Table:** 

A. Grantee Identifier Number	No. of FITs	% of Positive FITs
3	188	0
17	1,011	5.0
2	2,475	5.1
12	1,156	5.7
4	5,334	6.2
16	400	6.3
24	109	6.4
28	3,197	6.8
18	452	8.0
21	1,989	8.6
14	399	9.5
13	2,620	9.6
25	255	12.2
10	3,031	10.4
26	480	13.3
7	54	13.0
8	148	14.9
27	1,663	25.1
All 28 grantees	24,983	8.7

**Table:** 

B. Grantee Identifier Number	No. of FOBTs	% of Positive FOBTs
16	813	0.7
15	3,092	3.6
11	3,978	5.0
28	136	6.6
14	34	8.8
26	762	11.5
13	4,299	13.0
All 28 grantees	13,157	7.4

The low positivity rate at grantee no. 3 for FIT and grantee no. 16 for FOBT cannot be explained by frequent rescreening of the same clients. These low rates persisted when we included only first stool tests in the CRCCP and excluded clients who reported screening before the program. The high FIT positivity rate at grantee no. 27 cannot be explained by a high proportion of clients with positive family or personal history of CRC. When these clients were excluded, the FIT positivity rate was nearly unchanged.

#### Completeness and timeliness of follow-up of positive tests

Overall, 82.9% (range by grantee, 50.0%–97.2%) of the 3,197 positive FOBT/FITs were followed up by diagnostic colonoscopy in the CRCCP; 1 in 6 (17.1%) were not followed up (range, 2.8%–50.0%) (Table 2). Of the 2,649 tests with follow-up, 79.8% had colonoscopy within 90 days of the positive stool test, just below the 80% quality indicator established for the CRCCP, and 15.4% had colonoscopy between 91 and 180 days; 95.2% of colonoscopies occurred within 180 days of the positive stool test. Our 80% quality indicator was not met at 5 of the 16 grantees with ≥30 positive tests.

**Table 2:** Completeness and Timeliness of Diagnostic Colonoscopy After a Positive Result From a Fecal Occult Blood Test or a Fecal Immunochemical Test in the Colorectal Cancer Control Program, by Grantee, 2009–2015^a^

Grantee Identifier^b^	No Colonoscopy Follow-Up, No. (%^c^) of Tests	Colonoscopy Follow-Up in . . ., No. (%^d^) of Tests			Total No. of Tests
		≤90 Days	91–180 Days	≥181 Days	
2	11 (8.7)	105 (90.5)	9 (7.8)	2 (1.7)	127
4	44 (13.2)	269 (92.8)	20 (6.9)	1 (0.3)	334
10	50 (15.9)	217 (81.9)	38 (14.3)	10 (3.8)	315
11	10 (5.1)	173 (92.0)	14 (7.4)	1 (0.5)	198
12	16 (24.2)	44 (88.0)	5 (10.0)	1 (2.0)	66
13	217 (26.4)	385 (63.7)	170 (28.1)	49 (8.1)	821
14	4 (9.8)	30 (81.1)	7 (18.9)	0	41
15	7 (6.4)	91 (88.3)	10 (9.7)	2 (1.9)	110
16	1 (2.8)	29 (82.9)	4 (11.4)	2 (5.7)	36
17	15 (29.4)	3 (8.3)	13 (36.1)	20 (55.6)	51
18	19 (50.0)	14 (73.7)	5 (26.3)	0	38
21	32 (18.7)	115 (82.7)	20 (14.4)	4 (2.9)	171
25	3 (8.8)	24 (77.4)	4 (12.9)	3 (9.7)	34
26	22 (13.8)	122 (88.4)	13 (9.4)	3 (2.2)	160
27	50 (12.0)	328 (89.1)	32 (8.7)	8 (2.2)	418
28	28 (12.2)	148 (73.3)	32 (15.8)	22 (10.9)	230
All 28 grantees^e^	548 (17.1)	2,113 (79.8)	407 (15.4)	129 (4.9)	3,197

a The Centers for Disease Control and Prevention provided Colorectal Cancer Control Program funding to grantees in 25 states and 4 tribal organizations for colorectal cancer screening, surveillance, and diagnostic services for underinsured or uninsured asymptomatic, low-income adults aged 50–64. One grantee was excluded from analysis because of missing data. Table shows data only for grantees (16 of 28) that had a total number of at least 30 positive tests (fecal occult blood tests or fecal immunochemical tests) during the program.

b Grantees identified by randomly assigned numbers.

c Percentages based on total N in row.

d Percentages based on the number of clients that had colonoscopy follow-up. Percentages may not sum to 100% because of rounding.

e Includes grantees that had fewer than 30 positive fecal occult blood tests or fecal immunochemical tests during the program.

Only grantee no. 7 provided sigmoidoscopy as the primary screening test to more than 10 clients. Of 492 sigmoidoscopies at this grantee, 96 (19.5%) were positive; of these, 76 (79.2%) were followed by colonoscopy, 63 (82.9%) of them within 90 days.

### Colonoscopy

Of the 27,612 colonoscopies performed in asymptomatic clients, the cecum was reached in 98.2%. The grantee-specific cecal intubation rate ranged from 94.2% to 100%, and was above 95% at all but 1 grantee. Bowel preparation quality was adequate in 97.9% of exams. The percentage of adequate preparation ranged from 93.0% to 99.6%.

#### Rescreening and surveillance recommendations after colonoscopy

A total of 20,928 average-risk clients had complete first colonoscopies in the CRCCP, either for primary screening or to follow up positive FOBTs or FITs. We excluded 853 of 20,928 (4.1%) clients because data on their screening outcome or recommended interval to the next test were incomplete. 

**Clients with a normal examination.** Of 20,075 average-risk clients, 11,192 (55.8%) had a normal outcome (ie, no polyps were found). Of these, 9,542 (85.3%) were told to return in 10 years for another colonoscopy (range among grantees, 37.7%–99.7%) as recommended in national guidelines (15), and another 9.2% (range among grantees, 0.3%–50.4%) were told to return in 5 years (Table 3, Appendix, Table A). A total of 242 (2.2%) of clients with a normal outcome were told to return for a test other than colonoscopy, usually an FOBT or FIT. At the 2 grantees with at least 30 clients who were told to have a stool test, 83% or more were told to have the test in 1 year, earlier than recommended.

**Table 3:** Rescreening and Surveillance Recommendations for Average-Risk Clients, by Outcome of the Initial Colonoscopy in the Colorectal Cancer Control Program, 2009–2015^a^

Initial Colonoscopy Outcome	Recommended Interval to Next Colonoscopy, No. (%) of Clients						Other Tests Recommended, No. (%) of Clients	Total No. of Clients (n = 20,075)
	<3 y	3 y	>3 y to <5 y	5 y	>5 y to <10 y	10 y		
Normal	64 (0.6)	99 (0.9)	5 (0)	1,035 (9.2)	205 (1.8)	9,542 (85.3)^b^	242 (2.2)	11,192
Hyperplastic or nonadenomatous polyps	65 (2.2)	149 (4.9)	7 (0.2)	677 (22.4)	75 (2.5)	1,961 (65.0)^b^^,^ c	85 (2.8)	3,019
1 or 2 Tubular adenomas <1 cm without high-grade dysplasia or villous histology	87 (2.9)	524 (17.5)	13 (0.4)	2,216 (74.1)^b^	15 (0.5)^b^	128 (4.3)^b^	6 (0.2)	2,989
Serrated polyps^d^	11 (4.9)	80 (35.6)	0	128 (56.9)	2 (0.9)	4 (1.8)	0	225
Advanced adenoma^e^	467 (18.6)	1,388 (55.2)^b^	8 (0.3)	624 (24.8)	4 (0.2)	11 (0.4)	14 (0.6)	2,516
>10 Adenomas of any size or histology	21 (42.0)^b^	20 (40.0)	0	9 (18.0)	0	0	0	50
Cancer	65 (77.4)^b^	8 (9.5)	0	5 (6.0)	0	1 (1.2)	5 (6.0)	84

a The Centers for Disease Control and Prevention provided Colorectal Cancer Control Program funding to grantees in 25 states and 4 tribal organizations for colorectal cancer screening, diagnostic, and surveillance services for underinsured or uninsured asymptomatic, low-income adults aged 50–64. One grantee was excluded from analysis because of missing data. Includes clients at average risk who underwent an initial complete colonoscopy as a primary screening test or to follow up a positive stool test. A total of 853 clients were excluded because of incomplete data on screening outcome or recommended interval to the next test.

b Intervals that adhere to national guidelines (15,16).

c Recommended surveillance interval for small (<1 cm) hyperplastic polyps in the rectum or sigmoid. Hyperplastic polyps proximal to the sigmoid may require earlier follow up (17).

d Recommended follow-up interval for serrated polyps depends on the location, size, number and histology of polyps (15,17).

e Advanced adenoma category includes findings of 3–10 adenomas of any size, ≥1 adenoma ≥1 cm, or ≥1 adenoma with villous histology or high-grade dysplasia.

**Clients with hyperplastic or other nonadenomatous polyps.** Of the 3,019 clients whose colonoscopies found only hyperplastic or other nonadenomatous polyps, 65.0% (range among grantees, 18.8%–96.0%) were told to return in 10 years for colonoscopy as recommended in national guidelines (15), 22.4% (range among grantees, 3.4%–51.9%) were told to return in 5 years, and 7.1% (range among grantees, 0%–29.0%) in 3 years or less (Table 3, Appendix, Table B). At the 1 grantee with at least 30 clients who were told to return for a stool test, all were told to have the stool test in 1 year.

**Clients with 1 or 2 small tubular adenomas.** Of the 2,989 clients who had only 1 or 2 small tubular adenomas, 4.3% (range among grantees, 0%–49.4%) were told to return in 10 years, and 74.1% (range among grantees, 32.1%–100%) in 5 years, both consistent with national guidelines that patients return in 5 to 10 years (15). A total of 20.4% (range among grantees, 0%–66.0%) were told to return in 3 years or less (Table 3, Appendix, Table C).

**Clients with advanced adenomas.** Of the 2,516 clients with advanced adenomas (3–10 adenomas, ≥1 adenoma ≥1 cm, or ≥1 adenoma with villous histology or high-grade dysplasia), 55.2% (range among grantees, 20.0%–84.6%) were told to return in 3 years as recommended in national guidelines (15), 24.8% (range among grantees, 3.8%–53.3%) in 5 years, and 15.3% (range among grantees, 0%–34.7%) within a year (Table 3, Appendix, Table D).

#### Adenoma detection rate

Overall, the ADR for average-risk clients who had colonoscopy as their primary screening test was 36.0% (range among grantees, 19.3%–54.5%) for men and 25.7% (range among grantees, 11.7%–43.3%) for women (Table 4). The ADR results were similar after excluding clients who reported prior screening.

**Table 4:** Adenoma Detection Rate^a^ Among Clients Aged ≥50 Years, by Grantee, Sex, and Reason for Test, Colorectal Cancer Control Program, 2009–2015^b^

Grantee Identifier^c^	Primary Screening (Average Risk of CRC)				Primary Screening (Family History of CRC)				Follow-Up to Positive FOBT/FIT			
	Male		Female		Male		Female		Male		Female	
	N	ADR	N	ADR	N	ADR	N	ADR	N	ADR	N	ADR
1	320	39.4	1,270	22.7	—	—	59	32.2	—	—	—	—
2	185	44.3	338	27.5	—	—	—	—	—	—	89	34.8
3	846	34.4	1,242	23.3	—	—	—	—	—		—	—
4	—	—	—	—	—	—	106	43.4	90	45.6	187	34.8
5	492	42.1	773	27.6	55	23.6	150	14.0	—	—	—	—
6	291	32.0	412	23.1	—	—	36	36.1	—	—	—	—
8	770	46.0	866	36.1	89	47.2	126	40.5	—	—	—	—
9	343	42.6	998	33.8			79	40.5	—	—	—	—
10	—	—	—	—	—	—	—	—	60	56.7	198	52.0
11	—	—	—	—	93	45.2	292	25.0	60	35.0	106	25.5
12	34	26.5	54	27.8			46	32.6			38	42.1
13			78	24.4	59	50.8	265	30.9	99	40.4	470	30.2
14	807	19.3	932	11.7	—	—	—	—	—	—	—	—
15	51	47.1	382	34.6	60	45.0	354	29.9			90	37.8
16	105	25.7	103	14.6	—	—	—	—	—	—	—	—
18	211	46.9	379	33.5	35	48.6	71	33.8	—	—	—	—
19	515	29.5	885	23.2	88	28.4	149	30.9	—	—	—	—
20	247	27.9	501	20.6	31	32.3	75	12.0	—	—	—	—
21	37	32.4	108	31.5	—	—	—	—	39	64.1	92	35.9
22	66	54.5	104	43.3	—	—	—	—	—	—	—	—
23	387	39.0	664	24.8	—	—	62	35.5	—	—	—	—
25	167	40.1	268	26.1	—	—	—	—	—	—	—	—
26	419	38.4	1,159	24.4	82	43.9	264	28.4	49	49.0	77	33.8
27	—	—	—	—	—	—	55	30.9	115	48.7	227	37.0
28	—	—	54	38.9	—	—	45	35.6	82	54.9	110	39.1
All 28 grantees	6,364	36.0	11,609	25.7	808	42.2	2,361	30.1	724	47.9	1,771	35.6

Abbreviations: —, grantee had fewer than 30 clients in category; ADR, adenoma detection rate; CRC, colorectal cancer; FOBT, fecal occult blood test; FIT, fecal immunochemical test.

a Defined as the percentage of clients with ≥1 adenoma detected.

b The Centers for Disease Control and Prevention provided Colorectal Cancer Control Program funding to grantees in 25 states and 4 tribal organizations for colorectal cancer screening, surveillance, and diagnostic services for underinsured or uninsured asymptomatic, low-income adults aged 50–64. One grantee was excluded from analysis because of missing data. Data are shown only for grantees that had at least 30 clients in the categories for sex and reason for test. Includes data on only the first colonoscopy obtained by each client in the program.

c Grantees identified by randomly assigned numbers.

The numbers of clients with positive family history who had screening colonoscopy and the numbers who had diagnostic colonoscopy after positive stool tests were small at most grantees, especially for men. Overall, the ADRs for screening colonoscopy for clients with family history of CRC were 42.2% for men and 30.1% for women. The ADRs for clients with diagnostic colonoscopy after positive stool tests or sigmoidoscopy were 47.9% for men and 35.6% for women.

## Discussion

Most of the quality indicators examined in our study were met at most grantees. Follow-up of positive stool tests took place within a reasonable amount of time for most grantees. Cecal intubation rates and bowel preparation quality were high at all grantees. Almost all grantees met recommended thresholds for ADRs (18). However, we found considerable variation in quality indicators, and some grantees fell short of desired levels for certain indicators.

Stool test positivity rates were higher or lower than expected at a few grantees. Positivity rates depend on population characteristics, including screening history, and test characteristics, including threshold values for positivity. Although only extra-sensitive FOBTs were used in the CRCCP, various FITs were used. Positivity rates should be monitored, and unusually high or low rates and changes in rates over time should be investigated to rule out problems with test kits or processing and to identify any need to improve client instructions.

Stool tests are effective only when patients with positive findings are followed up with colonoscopy. In the CRCCP, 82.9% of positive results were followed up with colonoscopy, below the 90% quality indicator originally set for the CRCCP but exceeding the 80% target recently set as a quality metric by the US Multi-Society Task Force (USMSTF) (19) and exceeding rates reported in many settings (20–22). Some of the apparent lack of follow-up might be due to follow-up outside the CRCCP.

Follow-up of positive stool tests with colonoscopy is known to be challenging. A recent systematic review of interventions to improve follow-up found that patient navigators and provider reminders or performance data may help improve follow-up rates (22).

Follow-up of positive stool tests occurred within a reasonable amount of time for most grantees. Of those with follow-up in the CRCCP, 79.8% had colonoscopy within 90 days of the positive stool test (just below the 80% quality indicator established for the CRCCP) and 15.4% had colonoscopy 91 to 180 days after a positive stool test. The United States has no consensus guidelines for the time interval between a positive stool test and follow-up colonoscopy. A recent large study of a community-based setting found no significant increase in risk of CRC or advanced-stage disease associated with colonoscopy follow-up within 10 months of a positive FIT compared with 8 to 30 days (23). Although disease progression may be slow in most people, a short target interval may heighten patients’ sense of urgency to follow up positive screening tests and reduce loss-to-follow-up due to patients moving or changing providers.

For colonoscopy, the specialty societies have proposed quality indicators for use in continuous quality improvement programs (4,5,18). To guide these efforts, the American Society for Gastrointestinal Endoscopy/American College of Gastroenterology Task Force on Quality in Endoscopy recommended a subset of 3 high-priority indicators: 1) ADR in asymptomatic average-risk persons (screening), 2) frequency of colonoscopies following recommended surveillance and rescreening intervals, and 3) cecal intubation rate with photodocumentation (5).

The cecal intubation rate was high for all grantees. The recommended performance target is 90% or more cecal intubation with photodocumentation for all examinations and 95% or more for screening examinations (5). In the CRCCP, the cecal intubation rate was more than 95% for all but 1 grantee, where the rate was 94.2%. We did not collect information on photodocumentation.

The ADR is generally considered the most important quality measure for colonoscopy. Its validity as a quality indicator was first demonstrated in a study of the Polish national colonoscopy screening program, in which ADRs were inversely related to the risk of interval CRC after screening colonoscopy (24). A larger study at Kaiser Permanente showed a dose-dependent inverse association between ADR and the risks of all-stage, advanced-stage, and fatal interval CRC (25). Recently, a prospective study of Poland’s national program found that improvement in ADR, achieved by a comprehensive quality assurance program, translated into reduced risks of interval cancer and CRC death after screening colonoscopy (26).

In 2006, the USMSTF recommended that ADRs in first-time screening examinations for people aged 50 or older should be at least 25% for men and 15% for women (18). In the CRCCP, these thresholds were met at all but 1 grantee for men and all but 2 grantees for women. In 2014, the USMSTF raised these targets to 30% for men and 20% for women (5). Five grantees had ADRs below the new target of 30% for men, and 2 grantees had ADRs below the new target of 20% for women.

Efforts to increase ADRs have met with mixed success (27). Some factors that may improve ADR include split-dose preparation, and provider education on flat and depressed lesions and on withdrawal technique and public reporting of ADR (5,28). Several studies have demonstrated improvement in ADR through regular feedback and monitoring (29).

We found deviations from recommended rescreening and surveillance intervals in both directions, as has been documented in other settings (11,30). For example, for clients with a normal colonoscopy, 1 in 10 were recommended to receive the next colonoscopy in 5 years or less. For clients with advanced adenomas, 1 in 4 were told to return in 5 or more years. Surveillance that occurs too frequently provides little or no benefit while exposing patients to the risk of complications, increasing costs, and wasting resources that could instead be used for primary screening. Waiting too long increases risk of disease progression to a point where treatment may be less effective.

Some clients were told to have a test other than colonoscopy, usually a stool test, as their next test. At a few grantees, most of these clients were told to return in 1 year for the stool test. Clients who have a negative colonoscopy may have a stool test as their next screening test, but it should be after a 10-year interval. Because the risk of advanced adenomas within a few years after negative findings is low, interval testing is discouraged (15).

In the CRCCP, endoscopists used their usual report formats and terminology, and site staff had to assign bowel preparation quality (adequate vs inadequate) based on the descriptors in the endoscopy report. Some of the recommendations to return sooner than indicated in the guidelines might reflect endoscopists’ concern that bowel preparation was suboptimal although classified as adequate in our database.

For hyperplastic polyps, the 10-year recommendation is for polyps 1 cm or less in the rectum or sigmoid colon. Hyperplastic polyps proximal to the sigmoid may warrant earlier return (17). Because we did not collect information about polyp location, we could not determine whether some of the recommendations for 5-year intervals were appropriate.

The quality measures discussed here were intended to measure the performance of individual endoscopists. However, we were able to look only at aggregated measures of performance at the grantee level. Poor performance by a clinician can be masked when data from large numbers of clinicians are combined. Variability among endoscopists is undoubtedly greater than variability among grantees. Screening programs and endoscopy practices should monitor performance at the level of the endoscopist so that improvement activities can be targeted to poor performers.

Under the Medicare Access and CHIP Reauthorization Act of 2015 (31), the Centers for Medicare & Medicaid Services (CMS) is required to implement a quality payment incentive program to reward value and outcomes (9). Clinicians, including those performing colonoscopy, may receive an increase or decrease in payments based on whether or not they participate in quality assessment. CMS is also moving toward public reporting of performance information to help consumers make informed choices about the health care they receive through Medicare.

Colonoscopy registries have been developed to facilitate monitoring. The GI Quality Improvement Consortium, a collaboration of the American Society for Gastrointestinal Endoscopy and American College of Gastroenterology, is a quality benchmarking registry for gastroenterology practices; it has more than 7.5 million colonoscopy cases as of January 2019 (8). Members submit data and receive reports that include the measures discussed here.

Even with the availability of funding, support services, and oversight provided by the federal screening program, CRCCP, we identified gaps in performance. Our findings reinforce the need for quality monitoring and improvement. Efforts to improve uptake that also monitor screening performance could achieve better patient outcomes. Enhanced education and feedback to providers on rescreening and surveillance guidelines may be needed in addition to expanded enrollment protocols to ensure that clients understand follow-up procedures.

## Appendix Supplemental Tables A–D

**Table A:** Rescreening and Surveillance Recommendations for Average-Risk Clients Receiving an Initial Colorectal Cancer Control Program Colonoscopy With an Outcome of Normal or No Findings, 2009–2015^a^

Grantee Identifier	Recommended Interval to Next Colonoscopy, No. (%) of Clients				Other Test Recommended, No. (%) of Clients	Total No. of Clients
	≤3 y	5 y	>5 y to <10 y	10 y		
1	5 (0.6)	48 (5.3)	22 (2.4)	827 (91.5)	2 (0.2)	904
2	13 (4)	14 (4.3)	4 (1.2)	291 (90.1)	1 (0.3)	323
3	46 (3.9)	171 (14.6)	43 (3.7)	913 (77.7)	2 (0.2)	1,175
4	0	15 (9.6)	0	139 (88.5)	3 (1.9)	157
5	7 (1.1)	34 (5.2)	2 (0.3)	611 (93.4)	0	654
6	3 (0.7)	27 (6.4)	1 (0.2)	388 (92.4)	1 (0.2)	420
8	11 (1.5)	108 (14.4)	11 (1.5)	622 (82.7)	0	752
9	7 (1)	91 (13.6)	12 (1.8)	560 (83.6)	0	670
10	0	7 (9.3)	1 (1.3)	63 (84)	4 (5.3)	75
11	1 (1.1)	2 (2.2)	0	62 (69.7)	24 (27)	89
12	9 (13)	26 (37.7)	1 (1.4)	26 (37.7)	7 (10.1)	69
13	5 (1.4)	20 (5.4)	11 (3)	217 (59)	115 (31.3)	368
14	4 (0.3)	30 (2.2)	5 (0.4)	1,298 (97.1)	0	1,337
15	10 (3.9)	130 (50.4)	15 (5.8)	102 (39.5)	0	258
16	15 (12)	43 (34.4)	4 (3.2)	57 (45.6)	4 (3.2)	125
18	6 (2.2	30 (11.2)	15 (5.6)	214 (80.1)	2 (0.7)	267
19	0	3 (0.3)	0	869 (99.7)	0	872
20	1 (0.2)	22 (4.5)	3 (0.6)	458 (94.2)	2 (0.4)	486
21	3 (2.4)	36 (28.6)	12 (9.5)	74 (58.7)	1 (0.8)	126
22	1 (1.7)	17 (28.8)	0	40 (67.8)	1 (1.7)	59
23	5 (0.9)	50 (9.1)	1 (0.2)	491 (89.8)	0	547
25	1 (0.4)	15 (5.8)	5 (1.9)	239 (91.9)	0	260
26	5 (0.5)	74 (8.1)	31 (3.4)	741 (81.3)	59 (6.5)	911
27	4 (2.6)	9 (5.8)	2 (1.3)	129 (82.7)	12 (7.7)	156
28	1 (0.9)	11 (9.9)	4 (3.6)	94 (84.7)	0	111
All 28 grantees	163 (1.5)	1,035 (9.2)	205 (1.8)	9,542 (85.3)	242 (2.2)	11,192

^a^ Grantee-specific data are displayed only for those grantees with at least 30 clients with this screening outcome. For some grantees, percentages do not add to 100% because recommendations for >3 year to <5 year intervals are not displayed because there were so few.

**Table B:** Rescreening and Surveillance Recommendations for Average-Risk Clients Receiving an Initial Colorectal Cancer Control Program Colonoscopy With an Outcome of Hyperplastic or Other Nonadenomatous Polyps, 2009–2015^a^

Grantee Identifier	Recommended Interval to Next Colonoscopy, No. (%) of Clients				Other Test Recommended, No. (%) of Clients	Total No. of Clients
	≤3 y	5 y	>5 y to <10 y	10 y		
1	12 (4.3)	51 (18.2)	10 (3.6)	204 (72.9)	2 (0.7)	280
2	6 (5.9)	15 (14.9)	0	80 (79.2)	0	101
3	40 (14.4)	84 (30.2)	4 (1.4)	150 (54)	0	278
5	8 (4)	40 (20)	2 (1)	150 (75)	0	200
6	3 (2.7)	15 (13.3)	0	94 (83.2)	1 (0.9)	113
8	19 (7.8)	56 (23)	7 (2.9)	160 (65.6)	1 (0.4)	244
9	10 (4.8)	44 (21)	1 (0.5)	155 (73.8)	0	210
10	5 (10.4)	20 (41.7)	0	23 (47.9)	0	48
13	9 (9.8)	16 (17.4)	7 (7.6)	33 (35.9)	27 (29.3)	92
14	4 (2.5)	13 (8.1)	3 (1.9)	140 (87.5)	0	160
15	18 (23.4)	40 (51.9)	3 (3.9)	16 (20.8)	0	77
16	20 (29)	33 (47.8)	1 (1.4)	13 (18.8)	1 (1.4)	69
18	9 (8.7)	39 (37.9)	12 (11.7)	43 (41.7)	0	103
19	1 (0.7)	5 (3.4)	0	143 (96)	0	149
20	0	8 (8.2)	0	90 (91.8)	0	98
21	2 (4.3)	23 (48.9)	3 (6.4)	19 (40.4)	0	47
22	3 (8.3)	17 (47.2)	1 (2.8)	13 (36.1)	2 (5.6)	36
23	4 (2.1)	41 (21.2)	2 (1)	146 (75.6)	0	193
25	3 (4.3)	9 (12.9)	4 (5.7)	52 (74.3)	1 (1.4)	70
26	23 (8.3)	73 (26.4)	13 (4.7)	123 (44.6)	41 (14.9)	276
27	4 (6.2)	18 (27.7)	0	42 (64.6)	1 (1.5)	65
28	2 (5.3)	4 (10.5)	2 (5.3)	30 (78.9)	0	38
All 28 grantees	214 (7.1)	677 (22.4)	75 (2.5)	1,961 (65)	85 (2.8)	3,019

^a^ Grantee-specific data are displayed only for those grantees with ≥30 clients with this screening outcome. For some grantees, percentages do not add to 100% because recommendations for >3 year to <5 year intervals are not displayed because there were so few.

**Table C:** Rescreening and Surveillance Recommendations for Average-Risk Clients Receiving an Initial Colorectal Cancer Control Program Colonoscopy With an Outcome of 1 or 2 Tubular Adenomas, 2009–2015^a^

Grantee Identifier	Recommended Interval to Next Colonoscopy, No. (%) of Clients				Total No. of Clients
	<3 y	3 y	5 y	10 y	
1	2 (1.2)	37 (22)	126 (75)	2 (1.2)	168
2	2 (2)	4 (4)	93 (93)	1 (1)	100
3	16 (5.3)	54 (17.9)	227 (75.4)	3 (1)	301
4	0	0	47 (100)	0	47
5	7 (2.7)	43 (16.5)	207 (79.3)	0	261
6	2 (1.9)	11 (10.5)	91 (86.7)	1 (1)	105
8	14 (4.6)	50 (16.4)	225 (73.8)	15 (4.9)	305
9	2 (0.8)	33 (12.4)	222 (83.5)	7 (2.6)	266
10	1 (2.6)	5 (12.8)	33 (84.6)	0	39
13	5 (4.8)	22 (21.2)	73 (70.2)	1 (1)	104
14	0	6 (4.2)	137 (95.8)	0	143
15	6 (5.2)	33 (28.7)	73 (63.5)	1 (0.9)	115
18	11 (9.2)	40 (33.6)	67 (56.3)	0	119
19	0	6 (3.3)	85 (47.2)	89 (49.4)	180
20	1 (0.9)	71 (65.1)	35 (32.1)	0	109
21	3 (7.5)	11 (27.5)	26 (65)	0	40
22	5 (11.9)	8 (19)	29 (69)	0	42
23	1 (0.9)	21 (18.4)	88 (77.2)	0	114
25	1 (1.4)	8 (11.1)	62 (86.1)	0	72
26	6 (3.1)	29 (15.1)	140 (72.9)	6 (3.1)	192
27	1 (2.2)	6 (13.3)	38 (84.4)	0	45
28	0	10 (17.9)	46 (82.1)	0	56
All 28 grantees	87 (2.9)	524 (17.5)	2,216 (74.1)	128 (4.3)	2,989

^a^ Grantee-specific data are displayed only for those grantees with ≥30 clients with this screening outcome. For some grantees, percentages do not add to 100% because recommendations for >3 year to <5 year intervals, >5 year to <10 year intervals, and other test recommended are not displayed because there were so few.

**Table D:** Rescreening and Surveillance Recommendations for Average-Risk Clients Receiving an Initial Colorectal Cancer Control Program Colonoscopy With an Outcome of Advanced Adenoma, 2009–2015^a,b^

Grantee Identifier	Recommended Interval to Next Colonoscopy, No. (%) of Clients				Total No. of Clients
	≤1 y	>1 y to <3 y	3 y	5 y	
1	14 (6.7)	3 (1.4)	87 (41.8)	100 (48.1)	208
2	22 (23.7)	0	52 (55.9)	18 (19.4)	93
3	35 (18.2)	5 (2.6)	101 (52.6)	51 (26.6)	192
4	0	0	42 (84)	8 (16)	50
5	27 (20.5)	6 (4.5)	94 (71.2)	5 (3.8)	132
6	11 (14.9)	0	60 (81.1)	3 (4.1)	74
7	9 (23.1)	0	20 (51.3)	10 (25.6)	39
8	55 (16.7)	13 (4)	191 (58.1)	67 (20.4)	329
9	13 (9.6)	1 (0.7)	97 (71.3)	24 (17.6)	136
10	9 (12)	3 (4)	37 (49.3)	26 (34.7)	75
13	13 (16.7)	2 (2.6)	56 (71.8)	5 (6.4)	78
14	4 (5.4)	0	52 (70.3)	18 (24.3)	74
15	25 (34.7)	9 (12.5)	29 (40.3)	8 (11.1)	72
16	4 (13.3)	1 (3.3)	7 (23.3)	16 (53.3)	30
18	18 (20.9)	7 (8.1)	39 (45.3)	22 (25.6)	86
19	11 (9.4)	0	95 (81.2)	9 (7.7)	117
20	4 (10.3)	0	33 (84.6)	2 (5.1)	39
21	19 (33.3)	6 (10.5)	21 (36.8)	11 (19.3)	57
22	13 (28.9)	1 (2.2)	9 (20)	20 (44.4)	45
23	23 (18.5)	3 (2.4)	54 (43.5)	43 (34.7)	124
25	2 (4)	5 (10)	33 (66)	8 (16)	50
26	17 (7.8)	9 (4.1)	75 (34.2)	107 (48.9)	219
27	15 (19.5)	2 (2.6)	27 (35.1)	30 (39)	77
28	14 (24.1)	1 (1.7)	40 (69)	3 (5.2)	58
All 28 grantees	386 (15.3)	81 (3.2)	1,388 (55.2)	624 (24.8)	2,516

^a^ Advanced adenoma includes findings of 3–10 adenomas of any size, ≥1 adenoma ≥1 cm, or ≥1 adenoma with villous features or high grade dysplasia.

^b^ Grantee-specific data are displayed for those grantees with ≥30 clients with this screening outcome. For some grantees, percentages do not add to 100% because recommendations for >3 year to <5 year intervals, >5 year intervals, and other test recommended are not displayed because there were so few.
